# Genotype-independent association between profound vitamin D deficiency and delayed sputum smear conversion in pulmonary tuberculosis

**DOI:** 10.1186/s12879-015-1018-5

**Published:** 2015-07-21

**Authors:** Kashaf Junaid, Abdul Rehman, Tahir Saeed, David A. Jolliffe, Kristie Wood, Adrian R. Martineau

**Affiliations:** Department of Microbiology and Molecular Genetics, University of the Punjab, Quaid-e-Azam Campus, PO Box No 54590, Lahore, 54000 Pakistan; Centre for Primary Care and Public Health, Barts and The London School of Medicine and Dentistry, Queen Mary University of London, London, E1 2AB UK; Gulab Devi Chest Hospital, Lahore, 54000 Pakistan; Genome Centre, Barts and The London School of Medicine and Dentistry, Queen Mary University of London, London, EC1M 6BQ UK

**Keywords:** Tuberculosis, Vitamin D deficiency, Vitamin D receptor, CYP2R1, Vitamin D binding protein, Single nucleotide polymorphism

## Abstract

**Background:**

Both vitamin D deficiency and genetic variants in the vitamin D receptor (VDR) have been reported to associate with delayed response to intensive-phase therapy for pulmonary tuberculosis. Studies investigating the influence of genetic variants in vitamin D binding protein (DBP) and vitamin D 25-hydroxylase (CYP2R1) on vitamin D status and response to antituberculous therapy are lacking.

**Methods:**

We conducted a longitudinal study in 260 patients initiating treatment for smear-positive pulmonary tuberculosis in Lahore, Pakistan. Vitamin D status and genotypes for polymorphisms in VDR (rs2228570, rs731236, rs1544410), DBP (rs7041, rs4588) and CYP2R1 (rs2060793, rs10500804, rs10766197) were determined at baseline. Sputum smear microscopy was performed at 2, 4, 6 and 8 weeks, and time to sputum smear conversion was estimated for each participant. Analyses were conducted to determine demographic, clinical and genetic determinants of baseline vitamin D status and time to sputum smear conversion.

**Results:**

Profound vitamin D deficiency (serum 25[OH]D < 25 nmol/L) was highly prevalent at TB diagnosis (present in 54 % of patients), and was independently associated with female vs. male sex (adjusted OR 2.60, 95 % CI 1.50 to 4.52, *P* = 0.001), recruitment in October to March inclusive (adjusted OR 1.75, 95 % CI 1.00 to 3.04, *P* = 0.047) and bilateral vs. unilateral disease (adjusted OR 1.89, 95 % CI 1.49 to 4.52 *P* = 0.025). Profound vitamin D deficiency was also independently associated with impaired response to antituberculous therapy (median time to sputum smear conversion 22.5 vs. 7.5 days for patients with serum 25[OH]D <25 nmol/L vs. ≥ 25 nmol/L, respectively; aHR 4.36, 95 % CI 3.25 to 6.65, *P* < 0.001). No polymorphisms in VDR, CYP2R1 and DBP studied associated with either baseline vitamin D status or time to sputum smear conversion.

**Conclusions:**

Profound vitamin D deficiency is very common among TB patients in Lahore, Pakistan, and is independently associated with significantly delayed sputum smear conversion. Polymorphisms in VDR, CYP2R1 and DBP did not associate with baseline vitamin D status or response to intensive-phase treatment in this patient group.

## Background

Despite widespread availability of antimicrobial therapy, tuberculosis continues to carry a significant case fatality rate, estimated at 4–7 % in notified HIV-uninfected cases [1]. An improved understanding of the risk factors for poor response to antituberculous therapy could lead to the development of new interventions to improve treatment outcome.

A growing body of evidence suggests that vitamin D deficiency may be one such factor [2]. High-dose vitamin D was used to treat tuberculosis in the pre-antibiotic era, [3] and vitamin D metabolites support the induction of a broad range of antimycobacterial effector responses *in vitro* [4–8]. Vitamin D deficiency associates with impaired response to antituberculous therapy [9–11]. Genetic variants in the vitamin D receptor (VDR), which transduces vitamin D signal to modify transcriptional responses, have also been reported to predict treatment response [12, 13]. Polymorphisms in the vitamin D binding protein (DBP), which transports vitamin D metabolites in the circulation, and the vitamin D 25-hydroxylase enzyme (CYP2R1), which converts ‘parent’ vitamin D to its major circulating metabolite 25-hydroxyvitamin D (25[OH]D), influence vitamin D status in the general population [14]. However, their potential influence on vitamin D status and response to antimicrobial therapy in TB patients has not previously been studied. Moreover, despite evidence that effects of vitamin D status on the response to *M. tuberculosis* are genotype-dependent, [15–17] studies investigating the potential effect of interactions between vitamin D status and polymorphisms in the vitamin D pathway on response to antituberculous therapy have not previously been performed. We therefore conducted an observational study to determine a) the prevalence and determinants of vitamin D deficiency among a cohort of patients with newly-diagnosed pulmonary tuberculosis in Lahore, Pakistan; b) whether baseline vitamin D status and / or polymorphisms in VDR, DBP or CYP2R1 associated with response to intensive-phase antituberculous therapy in these patients as main effects, and c) whether any associations between vitamin D deficiency and response to treatment were genotype-dependent.

## Methods

### Study population

Patients attending Gulab Devi Chest Hospital, Lahore, Pakistan (Latitude 31.5° N) for treatment of smear-positive pulmonary tuberculosis were screened for eligibility to participate in the study. The following were excluded: those aged less than 14 years or more than 60 years; those who had already initiated antituberculous therapy; those with a medical record diagnosis of type 1 or type 2 diabetes mellitus, chronic renal failure, hepatic failure or ischaemic heart disease; and those with serological evidence of Hepatitis B, Hepatitis C or HIV infection. Those fulfilling eligibility criteria completed a questionnaire detailing age, gender and socioeconomic status (as indicated by whether or not they were in receipt of financial aid for hospital costs). All participants had a chest radiograph at baseline, which was classified as showing either unilateral or bilateral disease by the consultant chest physician in charge of their care. Participants also gave a sputum sample (for microscopy +/− mycobacterial culture as detailed below) and a 5 ml blood sample at baseline. Half of this blood sample was drawn into a serum tube, and the half was drawn into an EDTA tube. Serum tubes were centrifuged, and serum was aspirated and stored at −20 °C for subsequent assay of 25(OH)D concentration. Whole blood in EDTA was stored at 4 °C for subsequent DNA extraction. Participants whose baseline blood sample was haemolysed were excluded from analyses. Participants then initiated directly-observed antituberculous treatment according to WHO guidelines [18] and were followed up 2, 4, 6 and 8 weeks later. At each time point they were asked to provide a repeat sputum sample for microscopy, until either their sputum sample was smear-negative, or they were unable to expectorate. Data from patients who attended their final follow-up visit late were included up to 10 weeks from initiation of antimicrobial treatment. Medical staff caring for participants were informed of their patients’ vitamin D status at the end of the study, and were free to manage this as they deemed appropriate after the participant had completed follow-up. The study was approved by the ethics committees of the School of Biological Sciences, University of Punjab (ref SBS 873–12) and of the Gulab Devi Chest Hospital, Lahore. All participants gave informed consent to take part.

### Laboratory methods

Microscopy of Ziehl-Nielsen (ZN) -stained sputum samples was performed in the Microbiology Laboratory at the Gulab Devi Chest Hospital by trained staff using written standard operating procedures. These staff were blinded to participants’ vitamin D status. Bacillary load in baseline sputum samples was classified according to the number of acid-fast bacilli seen per high power field (HPF). Mycobacterial culture and drug sensitivity testing was done for a sub-group of patients who fulfilled hospital criteria for performing this test, namely those who were sputum-smear positive after 2 weeks of intensive-phase anti-TB treatment, and who had either been previously treated for tuberculosis or who were known to have had contact with patients with confirmed MDR-TB. Sputum samples from patients selected for drug sensitivity testing were initially assessed using the GeneXpert MTB/RIF platform [19]. Samples which were positive on this molecular test were then cultured on Lowenstein Jensen medium, and colonies were sent for phenotypic drug sensitivity testing using the Bactec MGIT system as described [20]. Serum 25(OH)D concentrations were determined by ELISA (Immunodiagnostic Systems, Boldon, UK); the inter-assay coefficient of variation was 11.5 %, and the average duration of sample storage prior to 25(OH)D assay was 91.2 days. Profound vitamin D deficiency was defined as a serum 25(OH)D concentration <25 nmol/L. Genomic DNA was extracted from whole blood [21] and quantified using the Nanodrop spectrophotometer and normalised to 5 ng/μl. 10 ng DNA was used as template for 2 μl TaqMan allelic discrimination assays (Applied Biosystems, Foster City, CA, USA) performed on the ABI 7900HT platform in 384-well format and analysed with Autocaller software, as previously described [22]. Pre-developed assays were used to type polymorphisms in genes encoding VDR (rs2228570 [FokI], rs731236 [TaqI] and rs1544410 [BsmI]), CYP2R1 (rs2060793, rs10500804 and rs10766197) and DBP (rs7041 [HaeIII] and rs4588 [StyI]). Genotyping assays failed in a small proportion of subjects, possibly as a result of poor DNA quality. Alleles at all loci conformed to the Hardy-Weinberg equilibrium.

### Sample size and statistical analyses

The study was prospectively powered to estimate prevalence of profound vitamin D deficiency in the study population with a 5 % error margin at the 95 % confidence level, using published algorithms [23]. Assuming an expected frequency of 79 %, [24] we calculated that a total of *n* = 253 participants would need to be recruited.

Statistical analyses were done with SPSS (version 20.0) and Stata IC (version 12) and figures were prepared with GraphPad Prism (version 4.00). The date of sputum smear conversion was estimated as the midpoint between the date of the last positive sputum smear and the date of the first negative sputum smear thereafter. Time to sputum smear conversion was calculated as the number of days from the date on which antituberculous treatment was initiated to the estimated date of sputum smear conversion.

Univariate analysis of associations between potential determinants of vitamin D status and risk of profound vitamin D deficiency was done using chi square tests; those found to associate with *P* < 0.1 on univariate analysis were included in multivariate analysis, which was done with binary logistic regression. Analysis of determinants of time to sputum smear conversion was done using Cox regression. Sub-group analyses to test for gene-environment interactions were performed by Cox regression with the inclusion of interaction terms between genotype and vitamin D status (25[OH]D < 25 nmol/L vs. ≥ 25 nmol/L); for these analyses, the Benjamini-Hochberg procedure for multiple testing correction was applied to control the false discovery rate with a q value threshold of 0.2 [25]. For all analyses, the influence of potential genetic determinants on dependent variables was assessed using additive models. Interaction effects were summarised as a ratio of hazard ratios with 95 % confidence interval and P-value. Statistical significance was assumed where *P* < 0.05.

## Results

### Participant enrolment and follow-up

**Fig. 1:**
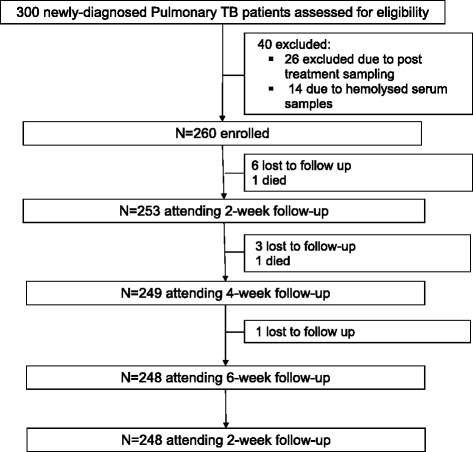
Participant enrolment and follow-up

Three hundred patients with smear-positive pulmonary TB fulfilling study eligibility criteria were recruited to the study from the Gulab Devi Chest Hospital, Lahore, Pakistan between August 2012 and November 2013. Of these, 40 were excluded from the analysis, 26 due to sampling after the start of antituberculous treatment and 14 due to haemolysis of blood samples. Of the remaining 260 patients, 248 completed follow-up, 10 were lost to follow-up and 2 died (Fig. 1).

### Baseline characteristics of study participants

The mean age of the 260 participants entering analysis was 31.6 years (standard deviation 10.5 years, range 14 to 55 years), and 140 (54 %) were female. One hundred and thirty seven patients (53 %) were enrolled in the sunnier part of the year (April to September inclusive), and 123 (47 %) were enrolled in the less sunny part of the year (October to March inclusive). One hundred and forty-four patients (55 %) had unilateral disease on chest radiograph, and 116 (45 %) had bilateral disease. Seventy-two patients (28 %) fulfilled hospital criteria for performing drug sensitivity testing, of whom 56 (78 %) had fully-sensitive disease and 16 (22 %) had MDR TB. Vitamin D deficiency was highly prevalent at baseline: 140 patients (54 %) had serum 25(OH)D < 25 nmol/L, 97 (37 %) had serum 25(OH)D 25–49.9 nmol/L, 17 (7 %) had serum 25(OH)D 50–74.9 nmol/L and only 6 (2 %) had serum 25(OH)D ≥75 nmol/L (Table 1). Median 25(OH)D concentration was 23.3 nmol/L (inter-quartile range 16.0 to 34.0 nmol/L).

**Table 1:** Demographic and clinical characteristics of study participants at baseline (*n* = 260)

Age	Mean age, years (s.d.)	.31.6 (10.5)
	Age range, years	14 to 55
Sex	Male, N (%)	120 (46 %)
	Female, N (%)	140 (54 %)
Socioeconomic status^a^	Lower, N (%)	177 (68 %)
	Higher, N (%)	83 (32 %)
Month of recruitment	April - September, N (%)	137 (53 %)
	October - March, N (%)	123 (47 %)
Sensitivity profile	Fully-sensitive	56 (22 %)
	Multidrug-resistant	16 (6 %)
	Undetermined	188 (72 %)
Extent of disease	Unilateral	144 (55 %)
	Bilateral	116 (45 %)
Sputum smear	1–99 AFB per 100 HPF	177 (68 %)
	≥100 AFB per 100 HPF	83 (32 %)
Serum 25-hydroxyvitamin D concentration	Median, nmol/L (IQR)	23.3 (16.0 to 34.0)
	<25 nmol/L, N (%)	140 (54 %)
	25.0 to 49.9 nmol/L, N (%)	97 (37 %)
	50.0 to 74.9 nmol/L, N (%)	17 (7 %)
	≥75.0 nmol/L, N (%)	6 (2 %)

*AFB* acid-fast bacilli, *HPF* high-power microscopy fields

^a^Lower socioeconomic status indicated by patient being in receipt of financial aid for hospital costs

### Determinants of baseline vitamin D status

**Fig. 2:**
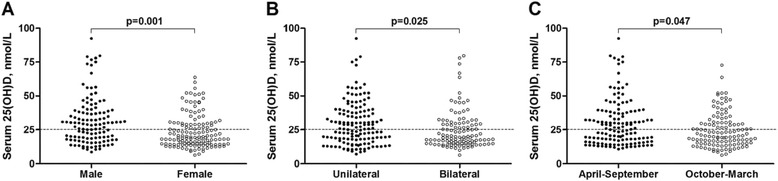
Serum 25-hydroxyvitamin D concentration for *n* = 260 study participants by sex (**a**), extent of disease (**b**) and season (**c**). P values from binary logistic regression adjusted for sex, extent of disease, season of enrolment and *BsmI* vitamin D receptor genotype

Phenotypic and genotypic determinants of baseline vitamin D status are presented in Table 2. Multivariate analysis revealed independent associations between profound vitamin D deficiency (serum 25[OH] D < 25 nmol/L) and female sex (aOR 2.60, 95 % CI 1.50 to 4.52, *P* = 0.001); recruitment between October and March, inclusive (aOR 1.75, 95 % CI 1.00 to 3.04. *P* = 0.047); and bilateral disease (aOR 1.89, 95 % CI 1.49 to 4.52, *P* = 0.025; Fig. 2). No independent associations were observed between baseline vitamin D status and age, socio-economic status, degree of smear-positivity at baseline or any polymorphism studied.

**Table 2:** Determinants of baseline Vitamin D status (*n* = 260)

					Univariate		Multivariate	
			N	25(OH)D <25 nmol/L, N (%)	OR (95 % CI)	P	Adjusted OR (95 % CI)^a^	P
Phenotypic characteristics	Sex	Male	120	49 (41 %)	Ref		Ref	
		Female	140	91 (65 %)	2.67 (1.62 to 2.45)	<0.001	2.60(1.50 to 4.52)	0.001
	Season of recruitment	Summer	137	66 (48 %)	Ref		Ref	
		Winter	123	74 (60 %)	1.62 (0.99 to 2.65)	0.053	1.75 (1.00 to 3.04)	0.047
	Socioeconomic status^b^	Lower	177	96 (54 %)	Ref			
		Higher	83	44 (53 %)	0.95 (0.56 to 1.60)	0.85	-	-
	Extent of disease	Unilateral	144	67 (46 %)	Ref		Ref	
		Bilateral	116	73 (63 %)	1.95 (1.18 to 3.21)	0.009	1.89 (1.49 to 4.52)	0.025
	Sputum smear	1–99 AFB per 100 HPF	106	53 (50 %)	Ref			
		≥100 AFB per 100 HPF	154	87 (56 %)	0.74 (0.45 to 1.22)	0.24	-	-
	Age	<30 years	124	67 (54 %)	Ref			
		≥30 years	136	73 (54 %)	0.98 (0.60 to 1.60)	0.95	-	-
Genotypic characteristics	rs731236, VDR	AA	83	38 (46 %)	Ref	0.14		
		AG	115	67 (58 %)	1.65 (0.93 to 2.92)		-	-
		GG	27	17 (63 %)	2.01 (0.82 to 4.91)		-	-
	rs1544410,VDR	CC	53	23 (43 %)	Ref	0.098		
		CT	128	74 (58 %)	1.78 (0.94 to 3.41)		1.66 (0.84 to 3.28)	0.14
		TT	54	34 (63 %)	2.21 (1.02 to 4.81)		2.23 (0.98 to 5.05)	0.054
	rs2228570,VDR	GG	137	76 (55 %)	Ref	0.96		
		GA	83	47 (57 %)	1.04 (0.60 to 1.81)		-	-
		AA	15	8 (53 %)	0.92 (0.31 to 2.67)		-	-
	rs2060793, CYP2R1	GG	112	62 (55 %)	Ref	0.81		
		AG	106	62 (58 %)	0.80 (0.22 to 2.94)		-	-
		AA	10	5 (50 %)	1.13 (0.66 to 1.94)		-	-
	rs10500804, CYP2R1	GG	59	34 (58 %)	Ref	0.34		
		GT	120	69 (58 %)	0.99 (0.53 to 1.87)		-	-
		TT	52	24 (46 %)	0.63 (0.29 to 1.33)		-	-
	rs10766197, CYP2R1	AA	52	29 (56 %)	Ref	0.60		
		AG	124	72 (58 %)	1.18 (0.57 to 2.11)		-	-
		GG	56	28 (50 %)	0.84 (0.37 to 1.72)		-	-
	rs7041, DBP	AA	55	34 (62 %)	Ref	0.57		
		AC	106	57 (54 %)	0.72 (0.37 to 1.47)		-	-
		CC	71	38 (54 %)	0.71 (0.34 to 1.45)		-	-
	rs4588,DBP	GG	122	66 (54 %)	Ref	0.14		
		GT	91	49 (54 %)	0.99 (0.57 to 1.70)		-	-
		TT	21	16 (76 %)	2.71 (0.93 to 7.88)		-	-

*25(OH)D* 25-hydroxyvitamin D, *OR* odds ratio, *Ref* referent category, *AFB* acid-fast bacilli, *HPF* high-power fields, *VDR* vitamin D receptor, *CYP2R1* vitamin D 25-hydroxylase, *DBP* vitamin D binding protein

^a^adjusted for sex, season of recruitment, extent of disease and rs1544410 genotype. ^b^lower socioeconomic status indicated by patient being in receipt of financial aid for hospital costs

**Fig. 3:**
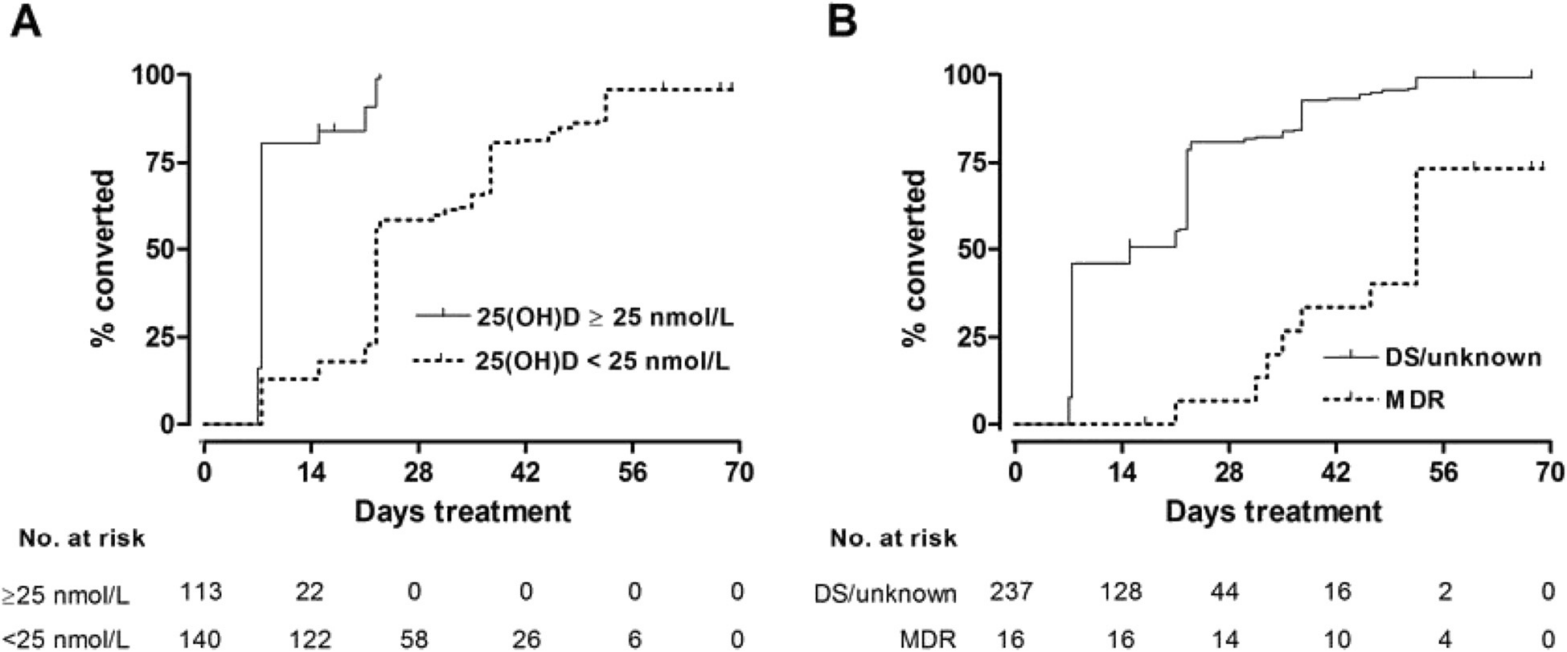
Time to sputum smear conversion by baseline vitamin D status (**a**) and drug sensitivity of *M. tuberculosis* isolate (**b**). Numbers of patients with positive sputum smear remaining in follow-up (number at risk) at 0, 14, 28, 42, 56 and 70 days are shown

### Determinants of time to sputum smear conversion

Phenotypic and genotypic determinants of time to sputum smear conversion are presented in Table 3. Multivariate analysis adjusting for sex, radiological extent of disease, baseline vitamin D status and drug sensitivity revealed independent associations between delayed sputum smear conversion and profound vitamin D deficiency at baseline (aHR 4.36, 95 % CI 3.25 to 6.65, *P* < 0.001). Isolation of a multidrug-resistant organism on sputum culture was also independently associated with delayed sputum smear conversion (aHR 3.35, 95 % CI 1.71 to 6.54, *P* < 0.001)(Fig. 3). No independent associations were observed between delayed sputum smear conversion and socio-economic status, radiographic extent of disease, age or any polymorphism investigated.

**Table 3:** Determinants of time to sputum smear conversion (*n* = 260)

					Univariate analysis		Multivariate analysis	
			N	Median TTSC, days (IQR)	HR (95 % CI)	P	Adjusted HR (95 % CI)^a^	P
Phenotypic characteristics	Sex	Male	120	7.5 (7.5–22.5)	1.47 (1.11–1.80)	0.01	1.16 (0.91–1.51)	0.26
		Female	140	22.5 (7.5–35)	Ref	-	Ref	-
	Socioeconomic status^b^	Lower	177	21 (7.5–30)	0.96 (0.73–1.23)	0.68	-	-
		Higher	83	21 (7.5–22.5)	Ref	-	-	-
	Extent of disease	Unilateral	144	7.5 (7.5–22.5)	1.38 (1.07–1.78)	0.01	1.21(0.85–1.55)	0.23
		Bilateral	116	22.5 (7.5–36.5)	Ref	-	Ref	-
	Sputum smear	1–99 AFB per 100 HPF	106	7.5 (7.5–22.5)	0.80 (0.6–1.01)	0.60	-	-
		≥100 AFB per 100 HPF	154	22.5 (7.5–23)	Ref	-	-	-
	Age	<30 years	124	21 (7.5–22.5)	1.14 (0.91–1.50)	0.33	-	-
		≥30 years	136	22 (7.5–37.5)	Ref	-	-	-
	25(OH)D	<25 nmol/l	140	22.5 (22.5–37.5)	4.93 (3.55–6.86)	<0.001	4.36(3.25–6.65)	<0.001
		≥25 nmol/l	120	7.5 (7.5–7.5)	Ref	-	Ref	-
	Drug sensitivity	Fully–sensitive / unknown	244	15 (7.5–22.5)	Ref	-	Ref	-
		Multidrug–resistant	16	52.5 (Undefined–35)	3.92 (2.12–7.24)	<0.001	3.35 (1.71–6.54)	<0.001
Genotypic characteristics	rs731236, VDR	AA	84	7.5 (7.5–22.5)	0.87 (0.71–1.06)	0.16	-	-
		AG	117	21 (7.5–30)				
		GG	29	22.5 (7.5–37.5)				
	rs1544410,VDR	CC	53	7.5 (7.5–22.5)	0.89 (0.73–1.08)	0.26	-	-
		CT	128	21 (7.5–35)				
		TT	54	21.5 (7.5–23)				
	rs2228570,VDR	GG	138	15 (7.5–23)	1.04 (0.84–1.28)	0.70	-	-
		GA	83	22.5 (7.5–22.5)				
		AA	15	7.5 (7.5–22.5)				
	rs2060793,CYP2R1	AA	10	21 (7.5–22.5)	0.88 (0.69–1.11)	0.28	-	-
		AG	106	21 (7.5–23)				
		GG	112	21 (7.5–23)				
	rs10500804,CYP2R1	GG	59	15 (7.5–22.5)	1.12(0.92–1.38)	0.24	-	-
		GT	120	22.5 (7.5–35)				
		TT	52	7.5 (7.5–22.5)				
	rs10766197,CYP2R1	AA	52	15 (7.5–22.5)	1.13 (0.92–1.38)	0.24	-	-
		AG	124	22.5 (7.5–35)				
		GG	56	15 (7.5–22.5)				
	rs7041,DBP	AA	55	22.5 (7.5–22.5)	1.0 (0.83–1.21)	0.94	-	-
		AC	106	15 (7.5–22.5)				
		CC	71	7.5 (7.5–23)				
	rs4588,DBP	GG	122	21 (7.5–23)	1.01 (0.82–1.23)	0.90	-	-
		GT	91	15 (7.5–22.5)				
		TT	21	22.5 (15–22.5)				

*TTSC* time to sputum smear conversion, *IQR* interquartile range, *HR* hazard ratio, *Ref* referent category, *AFB* acid–fast bacilli, *HPF* high–power microscopy fields, *25(OH)D* 25–hydroxyvitamin D, *VDR* vitamin D receptor, *CYP2R1* vitamin D 25–hydroxylase, *DBP* vitamin D binding protein

^a^adjusted for sex, extent of disease, baseline vitamin D status and drug sensitivity; ^b^lower socioeconomic status indicated by patient being in receipt of financial aid for hospital costs

In order to investigate the possibility that the presence of undiagnosed MDR-TB may have confounded the relationship between low baseline vitamin D status and delayed sputum culture conversion, we repeated the multivariate Cox regression analysis of determinants of time to sputum smear conversion in the sub-group of 72 patients for whom DST results were available, adjusting for sex, radiological extent of disease, baseline vitamin D status and drug sensitivity as before. The independent association between low baseline vitamin D status and delayed sputum smear conversion remained strong after adjustment for sex, radiological extent of disease and drug sensitivity (adjusted Hazard Ratio [aHR] 4.51, 95 % CI 1.91 to 10.70, *P* = 0.001). The independent association between presence of MDR TB and delayed sputum smear conversion was also replicated in this sub-group analysis (aHR 3.59, 95 % CI 1.77 to 7.30, *P* < 0.001). The findings of this sub-group analysis suggest that the association between low vitamin D status and delayed sputum smear conversion seen in the study population as a whole are likely to be independent of the effects of MDR TB on this outcome.

### Influence of gene-environment interaction on time to sputum conversion

We have previously reported that polymorphisms in the vitamin D receptor modify the effect of vitamin D supplementation on time to sputum culture conversion [17]. We were therefore interested to determine whether the strength of association between profound vitamin D deficiency and delayed sputum smear conversion differed according to genetic variation in VDR, DBP and CYP2R1. Results of the pertinent interaction analyses are presented in Table 4. Prior to correction for multiple analyses, statistically significant associations between profound vitamin D deficiency and delayed sputum smear conversion were found in patients with the GG genotype of rs2228570,VDR (adjusted HR 6.53, 95 % CI 3.99 to 10.66, *P* < 0.001) and in those with the GA genotype (adjusted HR 3.02, 95 % CI 1.82 to 5.01, *P* < 0.001) but not in those with the AA genotype (adjusted HR 4.58, 95 % CI 0.56 to 37.25, *P* = 0.16; P for interaction = 0.03). However, this interaction did not attain statistical significance after correction for multiple analyses. P values for the interaction terms for all other genotypes were >0.05.

**Table 4:** Influence of vitamin D status on time to sputum smear conversion by genetic sub–groups

SNP, Gene	Genotype	N	Adjusted HR for 25(OH)D <25 vs. ≥25 nmol/L (95 % CI)^a^	P	HR, interaction term (95 % CI)	P_interaction_^b^
rs731236, VDR	AA	84	5.87 (3.20–10.76)	<0.001	0.84 (0.56–1.24)	0.37
	AG	117	4.27 (2.65–6.86)	<0.001		
	GG	29	5.53 (1.92–15.94)	<0.001		
rs1544410,VDR	CC	53	5.97 (2.73–13.04)	<0.001	0.79 (0.53–1.17)	0.24
	CT	128	4.45 (2.81–7.05)	<0.001		
	TT	54	4.61 (2.25–9.43)	<0.001		
rs2228570,VDR	GG	138	6.53 (3.99–10.66)	<0.001	0.61 (0.38–0.96)	0.03
	GA	83	3.02 (1.82–5.01)	<0.001		
	AA	15	4.58 (0.56–37.25)	0.16		
rs7041,DBP	AA	55	4.81 (2.49–9.32)	<0.001	1.0 (0.69–1.45)	0.98
	AC	106	6.39 (3.61–11.30)	<0.001		
	CC	71	3.29 (1.86–5.83)	<0.001		
rs4588,DBP	GG	122	3.65 (2.32–5.75)	<0.001	1.09 (0.70–1.68)	0.69
	GT	91	6.46 (3.60–11.59)	<0.001		
	TT	21	3.92 (3.60–5.78)	<0.001		
rs10500804,CYP2R1	GG	59	4.46 (2.28–8.71)	<0.001	1.06 (0.71–1.58)	0.76
	GT	120	4.69 (2.89–7.61)	<0.001		
	TT	52	5.38 (2.35–12.31)	<0.001		
rs2060793,CYP2R1	AA	10	4.47 (0.81–24.47)	0.084	0.86 (0.54–1.36)	0.53
	AG	106	6.79 (3.86–11.94)	<0.001		
	GG	112	4.10 (2.52–6.68)	<0.001		
rs10766197,CYP2R1	AA	52	4.15 (2.08–8.29)	<0.001	1.03 (0.68–1.54)	0.88
	AG	124	5.12 (3.15–8.32)	<0.001		
	GG	56	4.82 (2.25–10.30)	<0.001		

*SNP* single nucleotide polymorphism, *HR* hazard ratio, *25(OH)D* 25–hydroxyvitamin D, *CI* confidence interval, *VDR* vitamin D receptor, *CYP2R1* vitamin D 25–hydroxylase, *DBP* vitamin D binding protein

^a^adjusted for drug sensitivity profile; ^b^none of these were significant using a Benjamini and Hochberg procedure controlling the false discovery rate at 20 %

## Discussion

To our knowledge, this is the first study to investigate potential interactions between vitamin D status and polymorphisms in the vitamin D pathway on the response to antituberculous therapy. We report that profound vitamin D deficiency is highly prevalent among newly-diagnosed TB patients in Pakistan, and that it is strongly and independently associated with markedly delayed sputum smear conversion. By contrast, polymorphisms in genes encoding VDR, DBP and CYP2R1 did not influence either vitamin D status or response to antituberculous therapy as main effects.

The high prevalence of profound vitamin D deficiency that we report among TB patients is striking, especially given that Lahore is located at 31.5° N, a latitude at which the intensity of ultraviolet radiation should be sufficient to induce cutaneous vitamin D synthesis year-round [26]. The mean serum 25(OH)D concentration among patients in our study (27.3 nmol/L) was lower than that reported in TB patients elsewhere in Punjab province, Pakistan (57.4 nmol/L), [27] and lower than that found among HIV-uninfected adults with active TB in Cape Town (40.5 nmol/L), [28] a sub-tropical setting that is approximately equidistant from the equator (latitude 33.9°S). In both of these case–control studies, vitamin D status was found to be lower among TB patients vs. controls. We hypothesise that low serum 25(OH)D concentrations may have preceded the onset of active TB, and impaired containment of latent TB infection, thereby leading to the development of active TB. This hypothesis is supported by findings of longitudinal studies conducted in Pakistan [24] and elsewhere [11] reporting that vitamin D deficiency in patients at risk of active TB precedes the development of active disease. However, reverse causality or residual confounding cannot be ruled out as explanations for this association, and trials of vitamin D for TB prevention are needed in order to determine whether vitamin D deficiency is a cause, rather than a consequence, of active TB. The co-existence of a high prevalence of vitamin D deficiency and high incidence of tuberculosis in sub-tropical settings such as Pakistan and South Africa makes them appropriate settings for such trials to be conducted. We also noted that vitamin D deficiency was more prevalent among female vs. male participants in our study; this may be attributable to sex differences in sun exposure and/or dietary vitamin D intake.

Our finding that vitamin D deficiency associates with impaired response to antituberculous therapy is consistent with reports from other observational studies to investigate this question. Rathored et al. reported an inverse association between serum 25(OH)D concentration and time to sputum smear conversion among 354 patients with multidrug-resistant tuberculosis in New Delhi, India [9]. Sato et al. reported a similar association among 34 patients with drug-sensitive disease in Fukushima, Japan [10]. More recently, Mehta et al. reported that vitamin D insufficiency (serum 25[OH]D <75 nmol/L) associated with increased risk of treatment relapse in a cohort of TB patients in Tanzania [11]. The consistency of this association in observational studies contrasts with findings of randomised controlled trials of adjunctive vitamin D to enhance response to antituberculous treatment, which have been more variable, [17, 29–32] although it is worth noting that studies utilising higher doses of vitamin D have reported acceleration of sputum smear conversion by around 2 weeks [29, 33] – similar to the 15 day difference in time to conversion reported in this study. Potential reasons for the discrepancy between observational studies and clinical trials could be explained by residual confounding in observational studies; inappropriate dosing regimens administered in clinical trials (doses either too small or too widely spaced to cause sustained elevation of serum 25[OH]D concentration); or insufficient power to detect potentially modest effects in drug-sensitive disease. At least three more trials with generous dosing regimens and/or larger sample sizes are in progress (ClinicalTrials.gov references NCT01657656, NCT01580007 and NCT00507000), and their results are awaited with interest.

Our finding that commonly-studied polymorphisms in the vitamin D receptor did not influence time to sputum smear conversion contrasts with studies that have variously reported associations with rs2228570 (FokI), [34] rs731236 (TaqI), [13, 34] and rs1544410 (BsmI) [9]. Moreover, we did not find any association between polymorphisms in CYP2R1 or DBP and treatment response. Based on our previous findings suggesting clinically important gene-environment interactions in this context [15–17] we went on to explore the possibility that genetic variation in the vitamin D pathway might modify the influence of vitamin D status on response to antituberculous treatment : sub-group analyses revealed the persistence of very strong associations between vitamin D deficiency and delayed smear conversion across genotypic sub-groups. Overall, our results suggest that the strong phenotypic influence of profound vitamin D deficiency appears to outweigh any more subtle effect of genotypic variation in the vitamin D pathway on response to antituberculous therapy.

Our study has several strengths: our investigation of the influence of gene-environment interactions in the vitamin D pathway on response to antituberculous therapy is novel, as is our investigation of polymorphisms in CYP2R1 and DBP. Loss to follow-up was low (<5 %) and antituberculous treatment was directly observed. Moreover, phenotypic and genotypic data were available to allow us to adjust for potential confounders of the relationship between vitamin D status and treatment response using multivariate analysis.

Our study also has some limitations. First, it was observational in nature; accordingly, residual confounding cannot be excluded as an explanation for the association between vitamin D deficiency and delayed smear conversion that we report. Second, information on time to sputum culture clearance was not available to us: culture is both more sensitive and more specific than microscopy for the detection of viable mycobacteria in sputum, and sputum culture conversion is the only outcome of intensive-phase treatment that has been shown to correlate with 2-year risk of relapse [35]. Third, drug sensitivity testing was not performed for all *M. tuberculosis* isolates, due to limited availability of resources. However, sensitivity testing was performed in patients at risk of drug-resistance (i.e. those who were sputum-smear positive after 2 weeks of intensive-phase anti-TB treatment, and who had either been previously treated for tuberculosis or who were known to have had contact with patients with confirmed MDR-TB). These criteria are used in day-to-day clinical practice at Gulab Devi Chest Hospital, and have proven to be sensitive for the identification of patients with drug-resistant tuberculosis: thus, there is a rationale for assuming that patients who did not fulfil these criteria are likely to have drug-sensitive disease.

## Conclusion

In conclusion, we report that profound vitamin D deficiency is highly prevalent among pulmonary TB patients in Lahore, Pakistan, and that this associates with markedly delayed sputum smear conversion in a genotype-independent manner. Our study adds to the growing body of literature pointing to inadequate vitamin D status as a correctable risk factor for delayed response to antituberculous therapy.
